# PdCu@rGO-based Electrochemical Sensor for Rapid Detection of Catechol

**DOI:** 10.3390/s26113550

**Published:** 2026-06-03

**Authors:** Xiaoying Shen, Muyu Yan, Qiongya Wan, Ming Li, Xuefeng Wang, Pengcheng Xu, Yongheng Zhu

**Affiliations:** 1 International Research Center for Food & Health, College of Food Science and Technology, Shanghai Ocean University, Shanghai 201306, China; sysshenpro@163.com (X.S.);; 2 State Key Laboratory of Transducer Technology, Shanghai Institute of Microsystem and Information Technology, Chinese Academy of Sciences, Shanghai 200050, China; 3 School of Microelectronic, University of Chinese Academy of Sciences, Beijing 100049, China

**Keywords:** Pd-Cu bimetallic electrocatalyst, graphene-based composite, pyrocatechol, sensing electrode, food safety

## Abstract

Catechol, a prevalent phenolic pollutant in food products, poses a significant threat to food safety, necessitating the development of rapid and sensitive detection methods. To overcome the limitations of conventional analytical techniques, such as expensive equipment and operational complexity, electrochemical sensors have gained considerable attention owing to their rapid response and facile miniaturization. However, the rational design of sensing materials that exhibit both high sensitivity and selectivity remains a significant challenge. Herein, a series of PdCu bimetallic nanoparticles supported on reduced graphene oxide (PdCu@rGO) composites with varying Pd/Cu molar ratios was synthesized via a one-step liquid-phase reduction method. Owing to the synergistic electronic effects between Pd and Cu and the high electrical conductivity of the rGO support, the resulting nanocomposites exhibited excellent electrocatalytic activity toward catechol oxidation. At the optimal Pd/Cu molar ratio of 1:2, the fabricated Pd_1_Cu_2_@rGO/SPE sensor demonstrated a broad linear range of 0.5–500 μM, a low limit of detection of 200 nM (S/N = 3), good repeatability (RSD = 4.9%), and robust anti-interference capability. Furthermore, the proposed sensor was successfully applied to the detection of catechol in spiked green tea and fruit juice samples without complex pretreatment, achieving satisfactory recoveries of 91.0–101.4% and 98.6–104.8%, respectively. This work provides a reliable platform for the rapid, on-site screening of catechol in food matrices and offers valuable experimental insights into the rational design of bimetallic alloy–graphene heterostructures.

## 1. Introduction

Catechol, a prevalent phenolic pollutant in food matrices, poses a severe threat to human health by damaging the nervous system, liver, and kidneys and has been classified as a priority control pollutant by the World Health Organization [1,2]. Consequently, the accurate and rapid detection of catechol is of paramount importance for health monitoring. Nanomaterial-based electrochemical sensing technologies have attracted considerable attention in food safety monitoring owing to their high sensitivity and rapid response [3]. Currently, catechol detection primarily relies on conventional laboratory-based techniques, such as chromatography and mass spectrometry [4,5]. However, these methods suffer from high instrumentation costs and tedious operational procedures, rendering them unsuitable for on-site rapid screening [6,7]. In contrast, electrochemical sensors offer compelling alternatives due to their fast response, cost-effectiveness, and facile miniaturization [8], wherein electrode-modifying materials serve as the core component for achieving efficient molecular recognition [9]. In recent years, novel carbon nanomaterials, such as two-dimensional graphene and its derivatives, have demonstrated immense potential in electrochemical sensing, owing to their unique geometric and electronic structures [10]. Nevertheless, pristine carbon materials typically exhibit drawbacks such as insufficient catalytic activity and poor selectivity, which severely limit the overall performance of the resulting sensors [11]. Consequently, decoration with noble metals is widely recognized as an effective approach to enhance sensing performance; however, the rational design and precise regulation of the composition and microstructure of these metal active centers remain core challenges in constructing high-performance electrochemical sensors [12,13].

Among various noble metal catalytic materials, palladium-based nanomaterials have been extensively utilized as critical active components in electrochemical sensors for detecting diverse analytes [14]. For instance, Pd nanoparticle-modified carbon materials enable highly sensitive detection of low-concentration phenolic pollutants, demonstrating their potential in trace substance recognition [15,16]. Nevertheless, in complex biochemical environments or real food matrices, the anti-interference capability of sensors is particularly critical for practical deployment. To address this challenge, bimetallic alloy structures have been developed to optimize sensing performance. For example, Feng et al. fabricated a PdAu alloy that exhibited a wide linear range and superior selectivity for the electrochemical detection of catechol [17]. Similarly, constructing bimetallic interfaces to induce interfacial electronic synergy has proven to be an effective strategy for enhancing sensor performance; for instance, a recently reported PdMo bimetallene sensor achieved highly sensitive dopamine detection owing to the electronic modulation between Pd and Mo [18].

Despite these advancements, several critical research gaps remain in the current literature. Although the PdCu bimetallic system offers obvious cost advantages and its nanoparticles have been extensively investigated in the field of heterogeneous catalysis [19], systematic studies on its electrochemical sensing performance toward catechol are still lacking. In particular, the underlying mechanism of how the Pd/Cu molar ratio modulates the electrocatalytic behavior remains ambiguous. Moreover, most existing studies are confined to idealized buffer solutions, lacking validation in complex, real food matrices such as green tea and fruit juice [20].

To address these gaps, the primary objective of this study is to rationally design PdCu bimetallic alloys to achieve highly sensitive and selective electrochemical detection of catechol. Accordingly, a series of PdCu@rGO composites with varying Pd/Cu molar ratios was synthesized via a facile, one-step liquid-phase reduction method [21] and subsequently modified onto disposable screen-printed electrodes (SPEs). Compared with conventional pristine noble metal sensors, the proposed PdCu@rGO system offers several distinct advantages; for instance, the partial substitution of Pd with lower-cost Cu significantly reduces material costs, the abundant Pd–Cu bimetallic interfaces provide additional active sites, and the electronic modulation of Pd by Cu optimizes the catalytic activity. Based on these considerations, we hypothesize that an appropriately Cu-rich PdCu composition can enhance the electron transfer efficiency from Cu to Pd, thereby imparting the system with optimal catalytic activity and selectivity. The final Pd_1_Cu_2_@rGO exhibited the best sensing performance for catechol. The sensor showed a wide linear range of 0.5–500 μM, a low detection limit of 200 nM (S/N = 3), excellent repeatability (RSD < 5%), and strong anti-interference capability. To meet the application requirements of food safety, the sensor is further applied to the spiked recovery detection of catechol in green tea and fruit juice beverages, achieving recoveries of 91.0–101.4% and 98.6–104.8%, respectively, with all RSD values below 5%. Through this design, we systematically elucidate the synergistic catalytic mechanism between Pd and Cu and comprehensively evaluate the sensor’s anti-interference capability and practical reliability in real food samples.

## 2. Materials and Methods

### 2.1. Chemicals and Reagents

Palladium(II) acetylacetonate (99%), ascorbic acid, Nafion solution (5 wt%), polyvinylpyrrolidone (PVP, M_w_ ≈ 30,000), and catechol (≥99%) were purchased from Sigma-Aldrich (St. Louis, MO, USA). Copper(II) acetate, anhydrous ethanol, ethylene glycol, ammonium chloride, urea, glucose, and sodium nitrite were of analytical grade and obtained from Sinopharm Chemical Reagent Co., Ltd. (Shanghai, China). Aspartame (≥98%) and sucralose (≥98%) were purchased from Shanghai Aladdin Biochemical Technology Co., Ltd. (Shanghai, China). Erythritol (≥99%) was acquired from Shanghai Macklin Biochemical Technology Co., Ltd. (Shanghai, China). Phosphate-buffered saline (PBS, 0.1 M, pH 7.0) was prepared in the laboratory. Ultrapure water (resistivity ≥ 18.2 MΩ·cm) was used throughout the experiments.

### 2.2. Instruments

Electrochemical measurements were performed on a CHI660E electrochemical workstation equipped with a CHI200B Faraday cage (Shanghai Chenhua Instrument Co., Ltd., Shanghai, China). A standard three-electrode configuration was adopted, comprising a screen-printed electrode (SPE) as the working electrode, a Ag/AgCl reference electrode, and a carbon counter electrode. Transmission electron microscopy (TEM) images were acquired on an FEI Tecnai G2 F20 microscope (FEI Company, Hillsboro, OR, USA) operating at an accelerating voltage of 200 kV. X-ray diffraction (XRD) patterns were recorded using a Bruker D2 Phaser diffractometer (Bruker, Billerica, MA, USA) with Cu Kα radiation (λ = 1.5406 Å) over a 2θ range of 5–90° at a scan rate of 2°/min. X-ray photoelectron spectroscopy (XPS) was conducted on a Thermo Scientific K-Alpha spectrometer (Thermo Fisher Scientific, Waltham, MA, USA) using a monochromatic Al Kα X-ray source (1486.6 eV). Inductively coupled plasma optical emission spectrometry (ICP-OES) was performed on a Thermo Fisher iCAP PRO system (Thermo Fisher Scientific, Waltham, MA, USA). Fourier-transform infrared (FTIR) spectra were recorded on a Thermo Fisher Nicolet iS50 spectrometer (Thermo Fisher Scientific, Waltham, MA, USA) within the range of 400–4000 cm^−1^. Raman spectra were collected using a Renishaw inVia Reflex Raman system (Renishaw plc, Wotton-under-Edge, UK) equipped with a 532 nm excitation laser over a wavenumber range of 100–3000 cm^−1^. The screen-printed three-electrode chips were provided by Shanghai University (Shanghai, China).

### 2.3. Preparation of PdCu Bimetallic Alloy

The PdCu@rGO composites were synthesized via a one-step liquid-phase reduction method. Palladium(II) acetylacetonate and copper(II) acetate served as the metal precursors, graphene oxide (GO) as the support, ethylene glycol as the solvent, anhydrous ethanol as the co-dispersant, ascorbic acid as the reducing agent, and PVP as the stabilizer. The Pd/Cu molar ratios were adjusted to 1:1, 1:2, and 2:1 by varying the amounts of the metal precursors while maintaining all other reaction parameters constant. In a typical procedure, target ratios of palladium(II) acetylacetonate and copper(II) acetate were dissolved in 5.0 mL of ethylene glycol under stirring (MYP11-2A magnetic stirrer, Shanghai Meiyingpu Instrument Manufacturing Co., Ltd., Shanghai, China) at room temperature for 30 min to ensure complete dissolution. Subsequently, 19.8 mg of GO, 50.0 mg of ascorbic acid, 86.0 mg of PVP, and 10.0 mL of anhydrous ethanol were sequentially introduced. Following 2 h of ultrasonic dispersion (KH-100 ultrasonic cleaner, Kunshan Hechuang Ultrasonic Instrument Co., Ltd., Kunshan, China), the mixture was transferred to an oil bath and reacted at 95 °C for 10 h (DF-101S magnetic stirrer, Gongyi Yuhua Instrument Co., Ltd., Gongyi, China). After cooling naturally to room temperature, the precipitate was isolated via centrifugation (TG16-WS centrifuge, Hunan Xiangyi Laboratory Instrument Development Co., Ltd., Changsha, China) and washed three to four times with an ethanol/deionized water mixture (1:1, *v*/*v*). Finally, the products were dried under vacuum at 65 °C for 8 h (DHG-9055A drying oven, Shanghai Yiheng Scientific Instruments Co., Ltd., Shanghai, China), yielding Pd_1_Cu_1_@rGO, Pd_1_Cu_2_@rGO, and Pd_2_Cu_1_@rGO, respectively [21]. For comparison, a control sample of Pd@rGO was prepared via an identical procedure.

### 2.4. Preparation of PdCu@rGO Modified Electrode

To prepare the modification suspension, 8 mg of PdCu@rGO powder was dispersed in 100 μL of anhydrous ethanol under ultrasonication for 5 min (KH-100 ultrasonic cleaner), followed by the addition of 25 μL of Nafion solution (5 wt%) and a further 2 min of ultrasonication. Subsequently, 1 μL of the resulting suspension was drop-cast onto the working electrode area of the SPE and allowed to dry naturally at room temperature to yield the PdCu@rGO-modified electrode. For comparison, GO-modified and Pd@rGO-modified electrodes were fabricated using the same procedure [22].

### 2.5. Electrochemical Measurements

A 10 mM catechol standard stock solution was prepared and serially diluted to the desired concentrations using PBS (0.1 M, pH 7.0) immediately prior to use. Cyclic voltammetry (CV) measurements were conducted within a potential window from −0.2 to +0.6 V (vs. Ag/AgCl) at a scan rate of 50 mV·s^−1^. Amperometric (i–t) measurements were performed at an applied potential of 0.28 V for 200 s with a sampling interval of 0.1 s. For electrochemical detection, 10 μL of the target solution was cast onto the electrode surface, and the measurement was initiated once the solution had completely wetted the electrode matrix.

### 2.6. Real Sample Testing

Commercially available green tea and fruit juice beverages were ultrasonically degassed for 5 min and filtered through a 0.22 μm membrane (Nylon, MilliporeSigma, Burlington, MA, USA). A 2.0 mL aliquot of the filtrate was diluted to 10 mL with PBS to serve as the real sample matrix. The standard addition method was employed by spiking the matrix with catechol standard solutions to achieve final spiked concentrations of 5, 50, and 100 μM. All electrochemical measurements were conducted in triplicate (*n* = 3) to evaluate the recovery and relative standard deviation (RSD) [23].

### 2.7. Statistical Analysis

The repeatability of the fabricated sensor was evaluated using five independently prepared electrodes (*n* = 5), and the data are expressed as the mean ± standard deviation (SD). The error bars in the corresponding figures represent the calculated SD. Precision was assessed by calculating the RSD, which was determined using the formula: RSD (%) = (SD/$\bar{X}$) × 100, where $\bar{X}$ is the mean value. The calibration curve was established via linear regression analysis, and the corresponding coefficient of determination (R^2^) was reported. For the spike recovery experiments in real samples, each concentration was evaluated in triplicate (*n* = 3). Data analysis and plotting were performed using OriginPro software (Version 2021, OriginLab Corporation, Northampton, MA, USA).

## 3. Results and Discussion

### 3.1. Material Characterization

**Figure 1:**
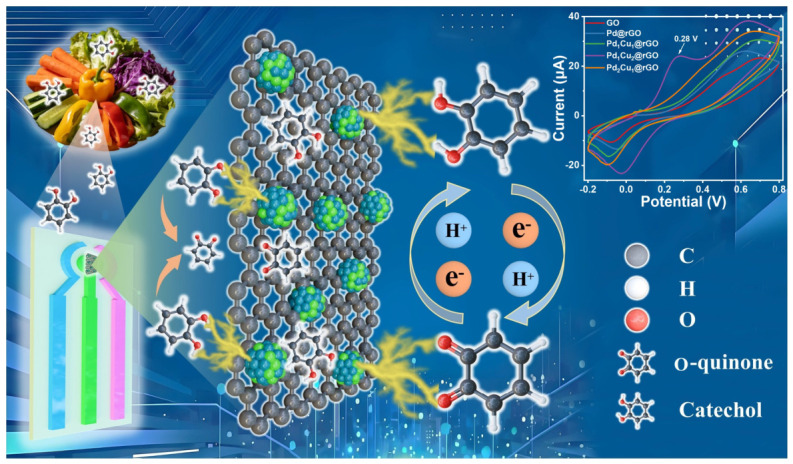
Schematic illustration of the fabrication of an electrochemical sensor based on a PdCu alloy supported on reduced graphene oxide for catechol detection.

**Figure 2:**
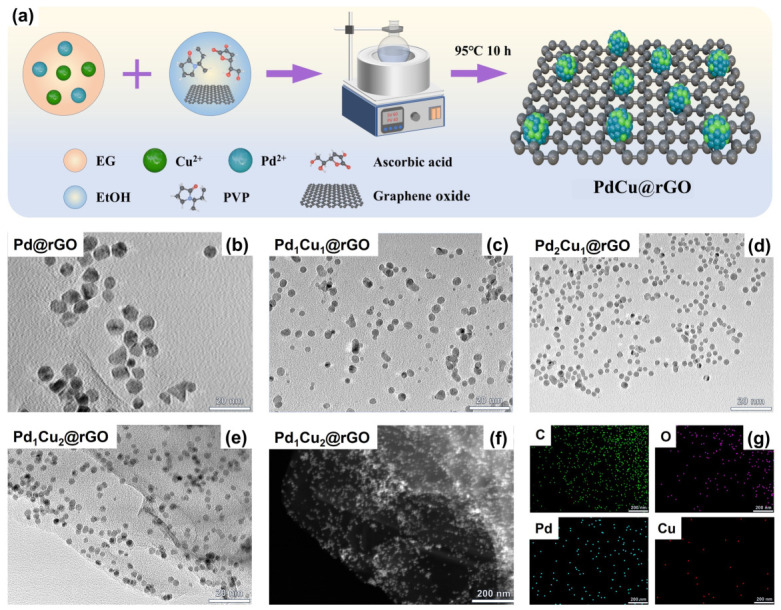
(**a**) Schematic illustration of the preparation process of PdCu@rGO composites via a one-step liquid-phase reduction method; (**b**) TEM image of Pd@rGO; (**c**) TEM images of Pd_1_Cu_1_@rGO, (**d**) Pd_2_Cu_1_@rGO, and (**e**) Pd_1_Cu_2_@rGO; (**f**) HAADF STEM image of Pd_1_Cu_2_@rGO; (**g**) corresponding EDS mapping of C, O, Cu, and Pd for the region shown in (**f**).

Figure 1 illustrates the overall design concept of this work, including the fabrication of the PdCu@rGO-modified electrode and the sensing mechanism for catechol. As shown in Figure 2a, a one-step liquid-phase reduction method [21] was employed to simultaneously achieve the reduction of graphene oxide (GO) to reduced graphene oxide (rGO) and the uniform deposition of PdCu alloy nanoparticles onto the rGO surface. By adjusting the Pd/Cu molar ratio, the micromorphology, crystal structure, and chemical valence states of the resulting alloy could be significantly regulated. Transmission electron microscopy (TEM) characterization revealed that the rGO retained an intact lamellar structure following the liquid-phase reduction (Figure S1). Compared to the Pd particles in the monometallic Pd@rGO (Figure 2b), the introduction of Cu resulted in a reduced size of the PdCu alloy nanoparticles, among which Pd_1_Cu_2_@rGO exhibited the smallest particle size and optimal dispersion (Figure 2c–e). The high-angle annular dark-field scanning transmission electron microscopy (HAADF-STEM) image of Pd_1_Cu_2_@rGO (Figure 2f) further confirmed the uniform size distribution of the nanoparticles. The corresponding energy-dispersive X-ray spectroscopy (EDS) elemental mapping (Figure 2g) demonstrated that C, O, Cu, and Pd were evenly distributed throughout the Pd_1_Cu_2_@rGO composite, with the Cu and Pd signals closely matching the spatial locations of the nanoparticles. This confirms the successful in situ growth of PdCu bimetallic nanoparticles on the rGO surface. Furthermore, inductively coupled plasma optical emission spectrometry (ICP-OES) measurements (Table S1) determined that the actual molar ratios of the Pd_1_Cu_1_@rGO, Pd_2_Cu_1_@rGO, and Pd_1_Cu_2_@rGO samples were 0.98:1, 1.85:1, and 0.68:1, respectively, demonstrating that the liquid-phase reduction method can effectively tune the bimetallic composition. Notably, the compositional deviation observed in Pd_1_Cu_2_@rGO (actual ratio 0.68:1) is due to the significantly higher standard reduction potential of Pd^2+^ (~+0.83 V) compared to Cu^2+^ (~+0.34 V) [24]. From a thermodynamic perspective, it is beneficial to preferentially reduce Pd^2+^, leading to the incomplete reduction of Cu^2+^ by ascorbic acid [25].

**Figure 3:**
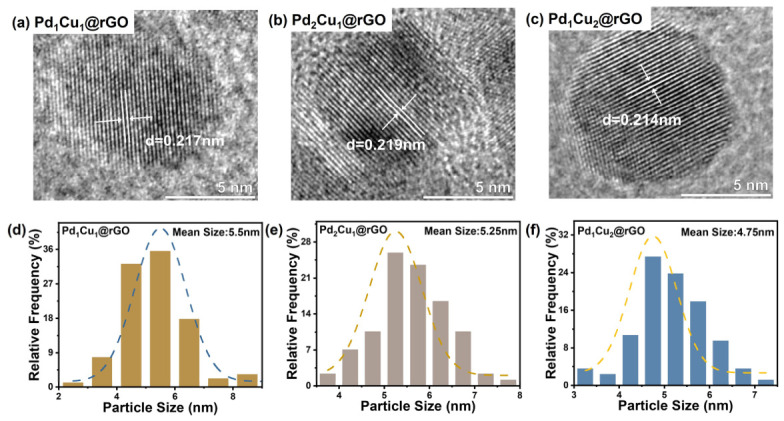
HRTEM images and particle size distributions of (**a**,**d**) Pd_1_Cu_1_@rGO, (**b**,**e**) Pd_2_Cu_1_@rGO, and (**c**,**f**) Pd_1_Cu_2_@rGO.

To further elucidate the crystal structure and particle size distribution of the PdCu@rGO composites, high-resolution transmission electron microscopy (HRTEM) characterization and statistical analysis of the particle sizes were performed, with the results presented in Figure 3. The HRTEM images (Figure 3a–c) revealed that the PdCu nanoparticles in Pd_1_Cu_1_@rGO, Pd_2_Cu_1_@rGO, and Pd_1_Cu_2_@rGO exhibited clear and continuous lattice fringes. Their respective interplanar spacings were measured to be 0.217 nm, 0.219 nm, and 0.214 nm, all of which fall exactly between the theoretical d-spacing of the pure Pd(111) plane (~0.225 nm) and the pure Cu(111) plane (~0.209 nm). This explicitly confirms that Pd and Cu formed a bimetallic alloy rather than a physical mixture [26]. Notably, the (111) facet is the most thermodynamically stable surface in face-centered cubic (FCC) metals. As widely recognized in electrocatalysis, this specific facet provides a stable and highly favorable geometric platform for the adsorption of aromatic molecules such as catechol [27]. Furthermore, the statistical analysis of the particle sizes (Figure 3d–f) indicated that the average nanoparticle diameters of Pd_1_Cu_1_@rGO, Pd_2_Cu_1_@rGO, and Pd_1_Cu_2_@rGO were 5.5 nm, 5.25 nm, and 4.75 nm, respectively. Among them, Pd_1_Cu_2_@rGO exhibited the smallest average particle size, the narrowest size distribution, and the best structural uniformity. Such ultrasmall and highly dispersed alloy nanostructures are highly conducive to exposing abundant catalytically active sites and providing shorter transport pathways for charge carriers [28,29].

**Figure 4:**
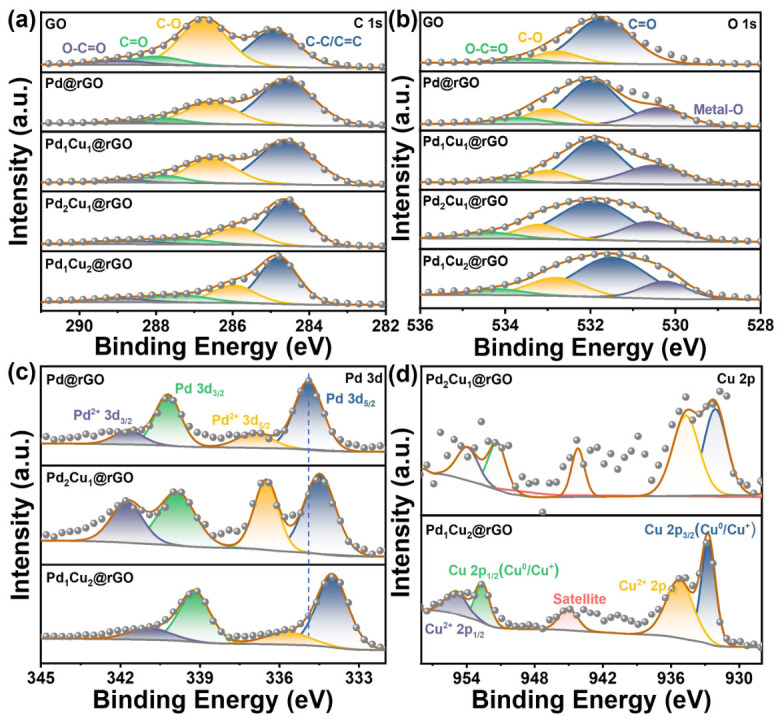
(**a**) High-resolution C 1s XPS spectra with peak-fitting deconvolution; (**b**) high-resolution O 1s XPS spectra with peak-fitting deconvolution; (**c**) high-resolution Pd 3d XPS spectra with peak-fitting deconvolution; (**d**) high-resolution Cu 2p XPS spectra with peak-fitting deconvolution. The samples include GO, Pd@rGO, Pd_1_Cu_1_@rGO, Pd_2_Cu_1_@rGO, and Pd_1_Cu_2_@rGO.

The X-ray photoelectron spectroscopy (XPS) survey spectra (Figure S2) revealed that GO contained only C and O elements, while Pd@rGO exhibited the characteristic Pd 3d peak. Furthermore, Pd_2_Cu_1_@rGO, Pd_1_Cu_1_@rGO, and Pd_1_Cu_2_@rGO simultaneously displayed the Pd 3d and Cu 2p peaks, confirming the successful loading of the bimetallic components [30]. The high-resolution C 1s spectra (Figure 4a) indicated that the GO surface contained abundant oxygen-containing functional groups, such as C–O and C=O. After the reduction and metal loading processes, the peak intensities of these oxygen-containing groups significantly decreased, accompanied by an increased proportion of the sp^2^-hybridized carbon skeleton (Table S2), demonstrating the effective reduction of GO to rGO [31]. Similarly, the O 1s spectra (Figure 4b) showed a reduction in the content of oxygen-containing groups and the emergence of a metal–oxygen interaction peak, suggesting a strong interfacial interaction between the metallic nanoparticles and the rGO support [32,33].

The high-resolution Pd 3d spectra (Figure 4c) were deconvoluted into two components corresponding to Pd^0^ and Pd^2+^. Compared with the monometallic Pd@rGO, the binding energies of Pd in the bimetallic samples shifted toward lower values, indicating electron transfer from Cu to Pd and confirming the formation of the PdCu alloy structure [34]. Specifically, as summarized in Table S3, the Pd^0^ 3d_5/2_ binding energies of Pd_2_Cu_1_@rGO and Pd_1_Cu_2_@rGO exhibited negative shifts of 0.4 eV and 0.8 eV, respectively, relative to Pd@rGO. This progressive shift magnitude clearly demonstrates that increasing the Cu content in the alloy induces a more pronounced electron transfer from Cu to Pd. Furthermore, the high-resolution Cu 2p spectra (Figure 4d) mainly consisted of Cu^0^/Cu^+^ species along with a minor amount of Cu^2+^, whose binding energies exhibited slight shifts with varying Pd/Cu ratios. This further corroborated the existence of a strong electronic synergistic effect between the two metals [35]. Such an optimized spatial electron distribution promotes the adsorption and activation of the target molecules in the electrochemical sensing process [36,37].

**Figure 5:**
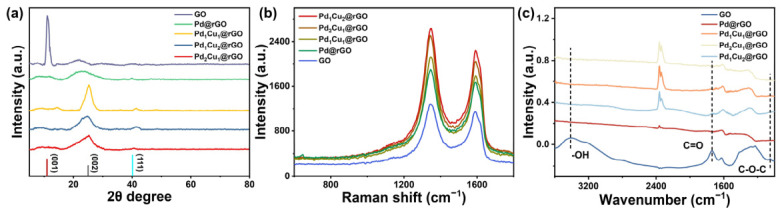
(**a**) XRD patterns of GO, Pd@rGO, Pd_1_Cu_1_@rGO, Pd_2_Cu_1_@rGO, and Pd_1_Cu_2_@rGO; (**b**) Raman spectra of all samples; (**c**) FTIR spectra of all samples.

X-ray diffraction (XRD), Raman spectroscopy, and Fourier-transform infrared (FTIR) characterizations were performed to systematically analyze the crystal structure, carbon skeleton orderliness, and surface functional group composition of the prepared samples. As shown in Figure 5a, pristine GO exhibited a sharp characteristic diffraction peak at ~10°, corresponding to its layered (001) structure. After the loading of the bimetallic PdCu components, this peak completely disappeared, and a broad (002) diffraction peak belonging to rGO emerged at ~25°, demonstrating that GO was successfully reduced to rGO during the metal loading process [38]. Furthermore, all PdCu@rGO samples displayed a characteristic diffraction peak at ~40°, which corresponds to the (111) crystal plane of the PdCu alloy. This result is highly consistent with the standard PDF card (PDF# 48-1551), confirming the successful synthesis of the PdCu alloy.

In the Raman spectra (Figure 5b), all samples exhibited characteristic peaks at ~1350 cm^−1^ (D band, corresponding to disordered carbon) and ~1580 cm^−1^ (G band, corresponding to the ordered sp^2^ carbon skeleton). The calculated I_D_/I_G_ ratios followed a descending order: GO (1.058) > Pd@rGO (0.982) > Pd_2_Cu_1_@rGO (0.968) > Pd_1_Cu_1_@rGO (0.947) > Pd_1_Cu_2_@rGO (0.904). This progressive decrease indicates that the graphitization degree of rGO was significantly enhanced upon metal loading, which corroborates the attenuated oxygen-containing peaks observed in XPS [39]. To obtain comprehensive structural insights, the exact absolute peak positions and the peak distance (Δω) between the D and G bands were extracted (Table S4). Notably, with the increasing proportion of Cu, the G band exhibited a prominent and continuous blue shift, moving from 1585.00 cm^−1^ in GO to 1592.69 cm^−1^ in Pd_1_Cu_2_@rGO. In carbon-based supported materials, such a G band blue shift is widely recognized as an indicator of interfacial charge transfer [40,41]. This phenomenon clearly demonstrates a strong electronic coupling between the bimetallic PdCu nanoparticles and the rGO support. This reinforced metal–support interaction not only improves the conjugated conductive network but also firmly anchors the nanoparticles to inhibit their agglomeration, perfectly complementing the excellent dispersion observed in TEM [42].

Finally, the FTIR spectra (Figure 5c) further verified the successful structural reconstruction of GO. In the PdCu@rGO samples, the characteristic peak intensities of oxygen-containing functional groups, such as –OH (~3400 cm^−1^), C=O (~1720 cm^−1^), and C–O–C (~1050 cm^−1^), were significantly weakened. This confirms the effective reduction of GO, while the residual oxygen-containing groups provided essential anchoring sites for the PdCu nanoparticles [43].

### 3.2. Electrochemical Sensing Performance and Mechanism

**Figure 6:**
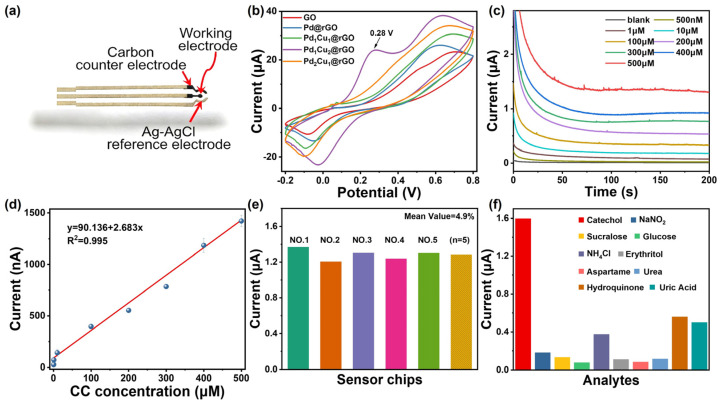
(**a**) Schematic diagram of the screen-printed three-electrode configuration; (**b**) CV curves of the bare electrode and various modified electrodes in 0.1 M PBS (pH 7.0) containing 10 mM catechol at a scan rate of 50 mV·s^−1^; (**c**) chronoamperometric response of the Pd_1_Cu_2_@rGO electrode to 0.5–500 μM catechol at 0.28 V; (**d**) linear fitting curve of the steady-state current versus catechol concentration (R^2^ = 0.995); (**e**) current responses of five independent electrodes to 400 μM catechol (RSD = 4.9%, *n* = 5); (**f**) selective responses to 500 μM catechol and various interferents (2 mM).

Figure 6 illustrates the electrochemical sensing performance of the PdCu@rGO-modified electrode toward catechol. Figure 6a presents a schematic diagram of the screen-printed three-electrode configuration, comprising a carbon working electrode, a carbon counter electrode, and a Ag/AgCl reference electrode. This miniaturized architecture provides robust hardware support for the rapid, on-site detection of catechol [44]. The cyclic voltammetry (CV) curves of the different modified electrodes (Figure 6b) revealed that the Pd_1_Cu_2_@rGO-modified electrode exhibited the highest oxidation peak current in a 10 mM catechol solution, with the oxidation peak potential negatively shifted to 0.28 V. Compared to the bare SPE, the modified electrode exhibits a significantly higher peak current and a lower overpotential, directly indicating its enhanced electrocatalytic activity toward catechol. To independently evaluate the interfacial electron transfer kinetics, cyclic voltammetry was performed in a 10 mM [Fe(CN)_6_]^3−^/^4−^ redox probe solution containing 0.1 M KCl (Figure S3). The Pd_1_Cu_2_@rGO/SPE electrode exhibited the highest peak current and the smallest peak-to-peak separation among all tested electrodes. The peak current strictly followed the order: Pd_1_Cu_2_@rGO > Pd_2_Cu_1_@rGO > Pd_1_Cu_1_@rGO > bare SPE. This trend explicitly confirms that the optimal Cu-rich bimetallic alloy significantly accelerates electron transfer, perfectly corroborating its superior electrocatalytic activity toward catechol.

At the optimal working potential of 0.28 V, the chronoamperometric (i–t) response curve of the Pd_1_Cu_2_@rGO-modified electrode (Figure 6c) demonstrated that the response current increased stepwise as the catechol concentration was gradually elevated from 0.5 μM to 500 μM. Moreover, a steady-state current plateau was rapidly reached at each concentration gradient, demonstrating fast response characteristics. Linear fitting of the steady-state response current versus the catechol concentration (Figure 6d) indicated an excellent linear relationship within the concentration range of 0.5–500 μM. The linear regression equation is expressed as *y* = 90.136 + 2.683*x* (where x represents the catechol concentration in μM and y represents the response current in nA), with a correlation coefficient (R^2^) of 0.995. Based on a signal-to-noise ratio of 3 (S/N = 3), the limit of detection (LOD) of this sensor was calculated to be 200 nM, proving its exceptional sensitivity and trace-level detection capability. As shown in Figure 6e, five independently prepared electrodes exhibited consistent current responses to 400 μM catechol, yielding a relative standard deviation (RSD) of 4.9%. This indicates excellent fabrication reproducibility. Although the sensor is fundamentally designed as a disposable device, its practical storage stability was evaluated over 14 days at 25 °C (Figure S4). The electrode retained 94.9%, 91.8%, and 85.2% of its initial current response after 3, 7, and 14 days, respectively, demonstrating satisfactory short-term storage capability for field deployment.

For the selectivity evaluation, nine common coexisting substances in food matrices (including inorganic ions, sugars, sweeteners, structurally similar phenols, and electroactive small molecules) were selected as potential interferents (Figure 6f). The results demonstrated that even when the concentration of the interferents was four times that of catechol (500 μM), the Pd_1_Cu_2_@rGO-modified electrode maintained a substantially high current response to catechol. Conversely, the response signals generated by the various interferents were negligible and comparable to the blank background. This confirms that the proposed sensor possesses exceptional selectivity and can effectively resist interference in complex food matrices, providing a reliable guarantee for precise detection in real samples.

To investigate the reaction kinetics, cyclic voltammograms of the Pd_1_Cu_2_@rGO/SPE were recorded at various scan rates (Figure S5a). As the scan rate increased, the oxidation peak current gradually augmented. By plotting the logarithm of the anodic peak current against the logarithm of the scan rate (Figure S5b), a strong linear relationship was obtained with a slope of 0.558 (R^2^ = 0.9976). According to the power-law equation *i_p_* = *a*·*v^b^*, a slope approaching 0.5 explicitly demonstrates that the electro-oxidation of catechol on this sensor is primarily a diffusion-controlled process [45].

The proposed sensor was compared with recently reported electrochemical sensors for catechol in the literature (Table 1). The results demonstrate that the Pd_1_Cu_2_@rGO/SPE sensor exhibits a broader linear range (0.5–500 μM) and a lower limit of detection (200 nM). Its overall performance is superior to that of most carbon-based or monometallic modified electrodes [46,47,48,49,50,51,52,53,54,55]. Concurrently, the fabrication approach based on screen-printed electrodes offers the distinct advantages of miniaturization and the elimination of the need for tedious electrode polishing. Compared with conventional electrode-modified sensors, it is significantly more suitable for rapid, on-site screening and possesses greater potential for practical applications. The superior performance resulting from the synergistic effect of the PdCu@rGO system can be delineated into three interconnected aspects. First, in this bimetallic system, Pd serves as the primary catalytic active center, while Cu acts as the crucial electronic modulator. This charge redistribution from Cu to Pd modulates the local electron density of Pd, which optimizes its binding affinity for catechol. Second, regarding geometric synergy, the Pd_1_Cu_2_@rGO possesses the smallest particle size (4.75 nm) and most uniform dispersion, providing maximum exposed active sites. Simultaneously, the exposed (111) facets place Pd and Cu atoms in intimate physical proximity, providing a stable and favorable geometric platform for catechol adsorption. Finally, regarding support synergy, Pd_1_Cu_2_@rGO exhibits the lowest I_D_/I_G_ ratio, indicating a highly restored graphitic network that ensures rapid electron transport. Therefore, the optimal activity of Pd_1_Cu_2_@rGO inherently arises from the synergistic combination of its ultrasmall size, maximized Cu-to-Pd electronic modulation, and highly conductive support.

### 3.3. Real Sample Testing in Green Tea and Fruit Juice

The Pd_1_Cu_2_@rGO sensor was employed for the spike-and-recovery analysis of catechol in commercially available green tea beverages and pure fruit juices (Table 2). No catechol was detected in either of the unspiked blank samples. Notably, real food matrices naturally contain complex polyphenolic compounds that could potentially interfere with electrochemical sensing. To effectively circumvent this issue, appropriate sample dilution was applied to minimize the initial matrix effect, followed by the standard addition method for precise quantification. By calculating the target analyte concentration based exclusively on the strictly linear current increments, this robust analytical strategy inherently compensates for any residual matrix background.

**Figure 7:**
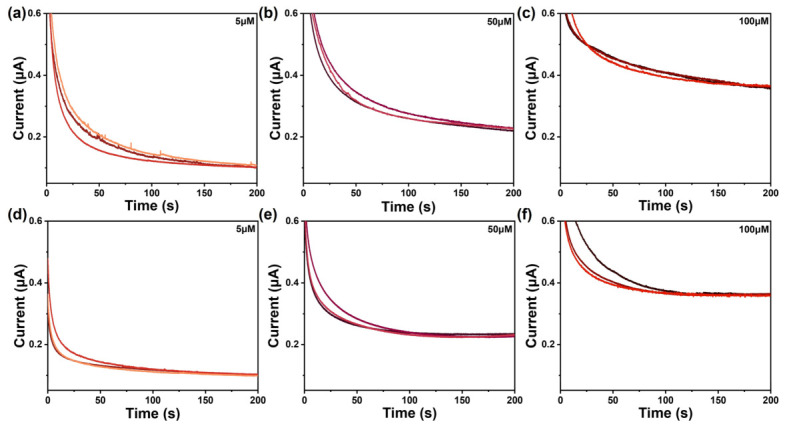
The i–t curves of green tea (**a**–**c**) and fruit juice (**d**–**f**) samples spiked with 5, 50, and 100 μM catechol, showing good reproducibility at each concentration.

The corresponding electrochemical response (i–t) curves for the spiked real samples are illustrated in Figure 7. For the green tea samples (Figure 7a–c), at three spiked concentration levels (5, 50, and 100 μM), the average recoveries were 91.0%, 101.4%, and 101.2%, respectively, with relative standard deviations (RSDs) ranging from 1.4% to 2.8%. Similarly, the fruit juice samples (Figure 7d–f) yielded average recoveries of 98.6%, 104.8%, and 101.1%, with RSDs of 2.9%, 2.3%, and 0.9%. All calculated RSD values were well below 5%. These excellent recovery results comprehensively demonstrate that, relying on the rational standard addition strategy, the proposed sensor enables highly accurate and reliable quantitative detection of catechol in complex food matrices without requiring complicated sample pretreatment.

## 4. Conclusions

In this study, a series of PdCu@rGO composites with tunable Pd/Cu molar ratios was successfully synthesized via a facile one-step liquid-phase reduction method and utilized to construct a disposable screen-printed electrochemical sensor for catechol detection. The comprehensive structural and electrochemical analyses reveal that the optimal Pd_1_Cu_2_@rGO composite possesses an ultrasmall particle size, uniform dispersion, and a highly conductive graphene network. More importantly, the explicit localized electron transfer from Cu to Pd profoundly modulates the surface electronic structure, giving rise to a strong bimetallic synergistic effect. Consequently, the optimized sensor exhibits significantly enhanced electrocatalytic activity, delivering a broad linear range, a low limit of detection, and exceptional selectivity. Furthermore, relying on a robust standard addition strategy, the sensor demonstrated highly accurate and reliable quantitative detection of catechol in complex real matrices (green tea and fruit juice) without complicated pretreatment. Ultimately, this work not only elucidates the structure–activity relationship underlying the bimetallic electronic synergy but also provides a highly promising and cost-effective strategy for developing high-performance disposable sensors for food safety monitoring.

## Figures and Tables

**Table 1:** Comparison of the analytical performance of the Pd_1_Cu_2_@rGO sensor with those of reported catechol sensors in the literature.

Electrode Material	Linear Range (μM)	LOD (μM)	Ref.
NMC/GCE	10–100	14	[46]
Co_3_O_4_/c-MWCNT/PolyVal/GCE	5–280	2.57	[47]
Poly(L-serine)/CPE	2–50	0.01	[48]
BC/Cu/CPE	10–100	1.03	[49]
ITO@TiO_2_/RGO/Pt	5–105	0.013	[50]
Sn MOF@rGO-650/GCE	0.20–28	0.033	[51]
PEDOT:PSS/IL/SPCE	0.1–330.0	23.7	[52]
Co_3_O_4_/MWCNTs/GCE	10.0–700.0	8.5	[53]
EGr/GC	0.6–100	0.182	[54]
Poly(NA) modified CPE	5–50	1.49	[55]
**Pd_1_Cu_2_@rGO/SPE**	**0.5–500**	**0.20**	**This work**

Note: The bold text highlights the performance of the materials synthesized in this work for clear comparison.

**Table 2:** Spiked recovery results of catechol in green tea and fruit juice samples using the Pd_1_Cu_2_@rGO sensor (*n* = 3).

Samples	Added/μM	Found/μM	Recovery/%	RSD/%
Green tea	0	Not found	-	-
	5	4.57	91.00	1.40
	50	50.70	101.40	2.80
	100	101.25	101.20	1.50
Juice	0	Not found	-	-
	5	4.93	98.6	2.90
	50	52.39	104.80	2.30
	100	101.11	101.10	0.90

## Data Availability

Data are contained within the article and Supplementary Materials.

## Supplementary Materials

The following supporting information can be downloaded at: https://www.mdpi.com/article/10.3390/s26113550/s1. Figure S1: (a) TEM image of GO; (b) TEM image of rGO after particle loading. Figure S2: XPS survey spectra of GO, Pd@rGO, Pd_1_Cu_1_@rGO, Pd_2_Cu_1_@rGO, and Pd_1_Cu_2_@rGO. Figure S3: Cyclic voltammograms of bare SPE, Pd_1_Cu_1_@rGO/SPE, Pd_2_Cu_1_@rGO/SPE, and Pd_1_Cu_2_@rGO/SPE in 10 mM [Fe(CN)_6_]^3−^/^4−^ solution containing 0.1 M KCl at a scan rate of 50 mV·s^−1^. Figure S4: Long-term storage stability of the Pd_1_Cu_2_@rGO/SPE electrode at a constant temperature (25 °C). The electrodes were stored for up to 14 days, and their current responses to 500 μM catechol were measured at days 1, 3, 7, and 14. Data are presented as normalized retention rate (%) relative to day 1 (mean ± SD, *n* = 2). Figure S5: (a) Cyclic voltammograms of the Pd_1_Cu_2_@rGO/SPE electrode in 10 mM catechol solution at scan rates of 10, 20, 50, 100, and 200 mV/s. (b) Corresponding plot of log(peak current) versus log(scan rate). The linear fit yields a slope of 0.558 and R^2^ = 0.9976, indicating a predominantly diffusion-controlled process. Table S1: ICP-OES results of Pd and Cu contents in the as-prepared samples (*n* = 3). Table S2: Compare the sp^2^ carbon skeletons and the total oxygen-containing functional group percentages of different samples. Table S3: Pd^0^ 3d_5/2_ binding energies of the Pd@rGO, Pd_2_Cu_1_@rGO and Pd_1_Cu_2_@rGO samples. Table S4: Detailed Raman peak positions, distances between G and D bands (Δω), and intensity ratios (I_D_/I_G_) for the prepared samples.
