# Supplementary material for: PdCu@rGO-based Electrochemical Sensor for Rapid Detection of Catechol
Source: Sensors (Basel). 2026 Jun 3;26(11):3550. doi: 10.3390/s26113550 (PMC13258880; doi:10.3390/s26113550)
Supplement: Supplementary file 1 [file sensors-26-03550-s001.zip › sensors-4331039-supplementary.pdf]

Supporting Information

PdCu@rGO-based Electrochemitry Sensors for Rapid and Protable Detection of Catechol

Xiaoying Shen [1,2], Muyu Yan [2,*] ,Qiongya Wan [1,2], Ming Li [2,3], XueFeng Wang [2,3], Pengcheng Xu [2,3], Yongheng Zhu[1,*]

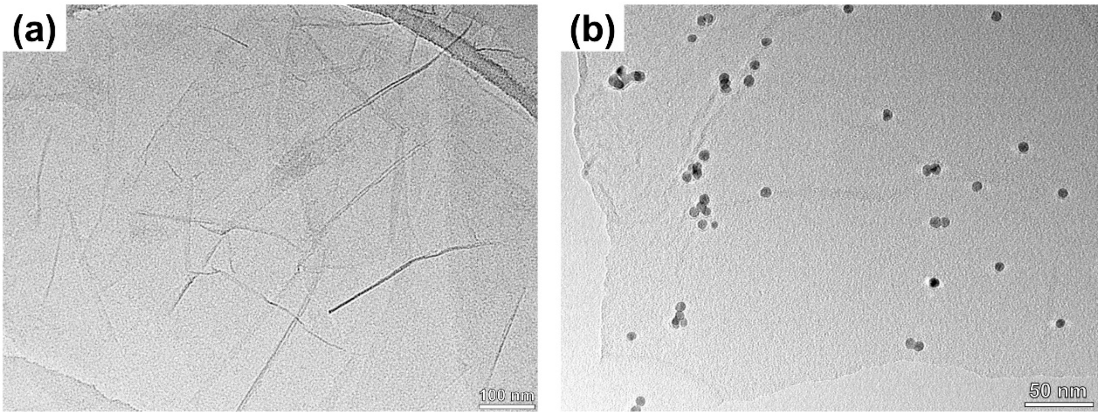

Figure S1. (a) TEM image of GO; (b) TEM image of rGO after particle loading.

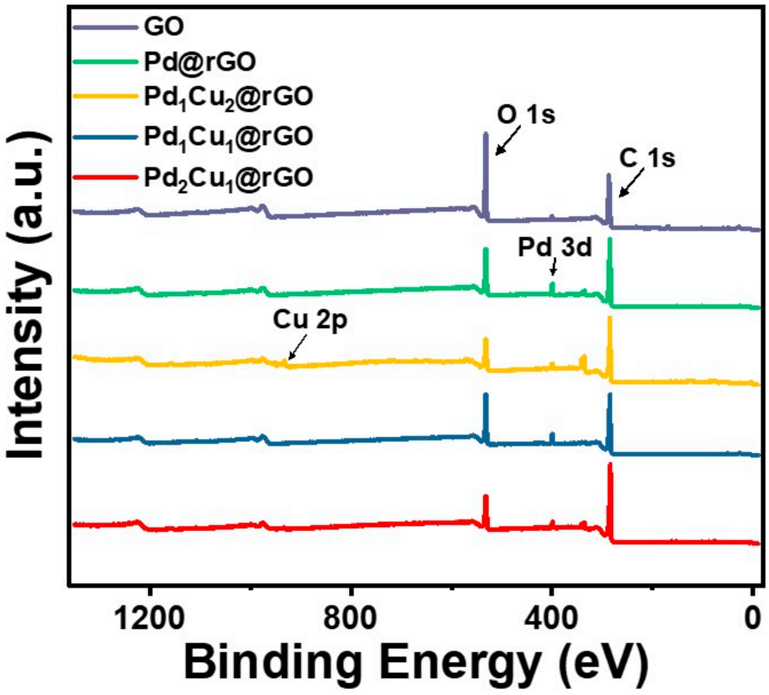

Figure S2. XPS survey spectra of GO, Pd@rGO, Pd_1Cu_1@rGO, Pd_2Cu_1@rGO, and Pd_1Cu_2@rGO.

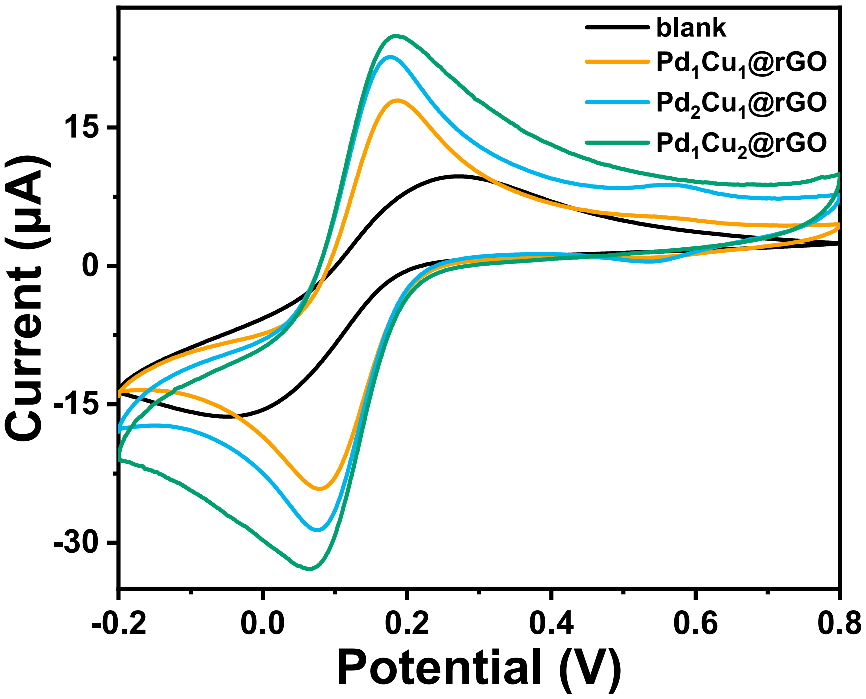

Figure S3. Cyclic voltammograms of bare SPE, Pd_1Cu_1@rGO/SPE, Pd_2Cu_1@rGO/SPE, and Pd_1Cu_2@rGO/SPE in 10 mM $[Fe(CN)_6]^{3-/4-}$ solution containing 0.1 M KCl at a scan rate of 50 mV·s^{-1}.

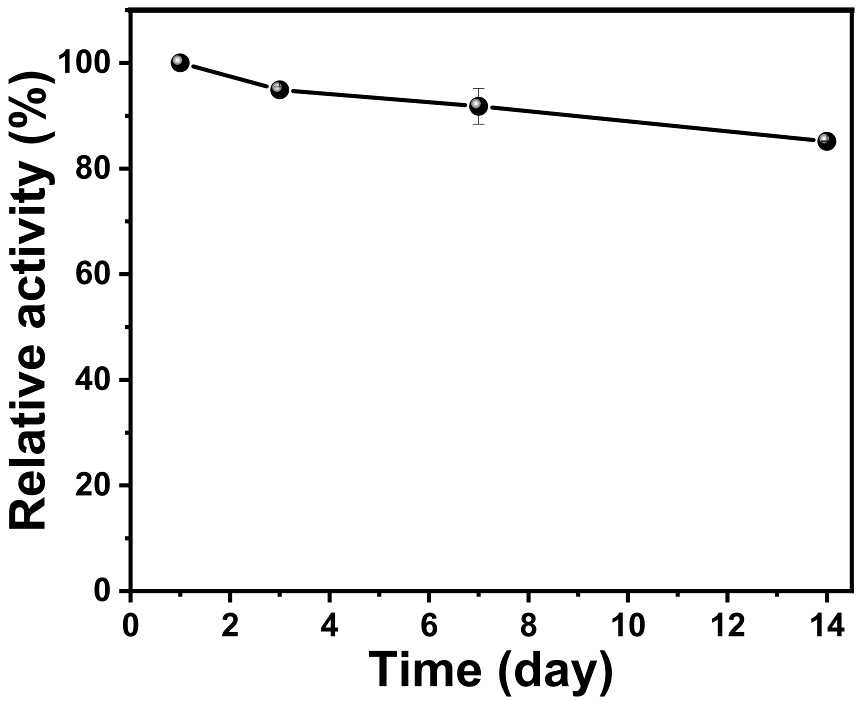

Figure S4. Long-term storage stability of the Pd_1Cu_2@rGO/SPE electrode at constant temperature (25 °C). The electrodes were stored for up to 14 days, and their current responses to 500 μM catechol were measured at days 1, 3, 7, and 14. Data are presented as normalized retention rate (%) relative to day 1 (mean ± SD, n = 2).

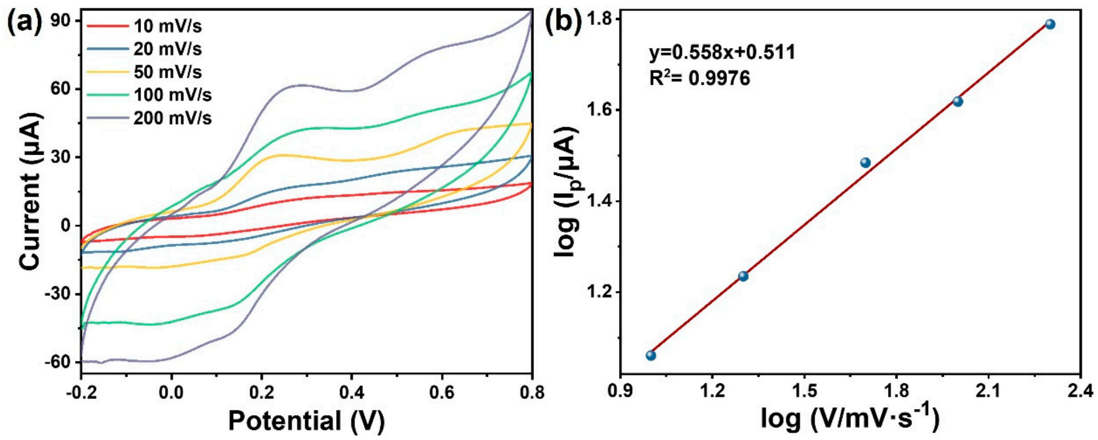

Figure S5.(a) Cyclic voltammograms of the Pd_1Cu_2@rGO/SPE electrode in 10 mM catechol solution at scan rates of 10, 20, 50, 100, and 200 mV/s. (b) Corresponding plot of log(peak current) versus log(scan rate). The linear fit yields a slope of 0.558 and R^2 = 0.9976, indicating a predominantly diffusion-controlled process.

Table S1. ICP-OES results of Pd and Cu contents in the as-prepared samples (n = 3).

Sample	Pd content（wt%）	Cu content（wt%）	Pd:Cu
Pd@rGO	10.76	-	-
Pd_1Cu_1@rGO	5.80	3.53	0.98:1
Pd_2Cu_1@rGO	7.71	2.49	1.85:1
Pd_1Cu_2@rGO	6.59	5.79	0.68:1

Table S2. Compare the sp^2 carbon skeletons and the total oxygen-containing functional group percentages of different samples.

Sample	sp^2 carbon framework	Total oxygen-containing functional groups
GO	43.52	56.49
Pd@rGO	59.80	40.20
Pd_1Cu_1@rGO	57.93	42.07
Pd_2Cu_1@rGO	52.00	48.00
**Pd_1Cu_2@rGO**	**57.93**	**42.07**

Table S3. Pd^0 $3d_{5/2}$ binding energies of the Pd@rGO, Pd_2Cu_1@rGO and Pd_1Cu_2@rGO samples.

Sample	Pd^0 $3d_{5/2}$ BE (eV)	ΔBE (vs. Pd@rGO) (eV)
Pd@rGO	334.9	0
Pd_2Cu_1@rGO	334.5	–0.4
Pd_1Cu_2@rGO	334.1	–0.8

Table S4. Detailed Raman peak positions, distances between G and D bands ($\Delta\omega$), and intensity ratios (I_D/I_G) for the prepared samples.

Sample	D band position (ωD, cm^{-1})	G band position (ωG, cm^{-1})	Distance ($\Delta\omega$, cm^{-1})	I_D/I_G
GO	1340.79	1585.00	244.21	1.058
Pd@rGO	1345.63	1588.00	242.37	0.982
Pd_2Cu_1@rGO	1344.42	1591.15	246.73	0.968
Pd_1Cu_1@rGO	1346.23	1592.10	245.87	0.947
Pd_1Cu_2@rGO	1348.65	1592.69	244.04	0.904
